# Development of Construction Workers Job Stress Scale to Study and the Relationship between Job Stress and Safety Behavior: An Empirical Study in Beijing

**DOI:** 10.3390/ijerph15112409

**Published:** 2018-10-30

**Authors:** Xiang Wu, Yuanlong Li, Yongzheng Yao, Xiaowei Luo, Xuhui He, Wenwen Yin

**Affiliations:** 1 School of Engineering and Technology, China University of Geosciences (Beijing), Beijing 100083, China; wuxiang@cugb.edu.cn (X.W.); 2102170092@cugb.edu.cn (Y.L.); HeXuhui@cugb.edu.cn (X.H.); 2 Key Laboratory of Deep GeoDrilling Technology, Ministry of Land and Resources, Beijing 100083, China; 3 School of Engineering Science, University of Science and Technology of China, Hefei 230026, China; yyz611@mail.ustc.edu.cn; 4 Department of Architecture and Civil Engineering, City University of Hong Kong, Hong Kong, China; 5 Department of Engineering Physics, Tsinghua University, Beijing 100084, China; yww17@mails.tsinghua.edu.cn

**Keywords:** construction worker, job stress scale, safety behavior, reliability analysis

## Abstract

Job stress is considered one of the critical causes of construction workers’ unsafe behaviors. As a mainstay industry in many countries, the construction industry has a considerable number of employees and the research on how job stress affects workers’ unsafe behaviors has important theoretical and practical significance to improve construction safety performance through better job stress management. In this study, the authors thoroughly reviewed the literature and conducted semi-structured interviews to identify the dimensions of job stress, designed the job stress scale and cited the safety behavior measurement scale. After that, a questionnaire survey was developed using the proposed measurement scale and distributed to the construction employees from a project in Beijing. One hundred fifty responses were collected and analyzed using reliability analysis to validate the scale’s internal consistency. Results from factor analysis indicate that the scales of job stress measurement can be grouped into six dimensions. To demonstrate the applicability of the developed scale on construction safety management research, the collected data was used to test the hypothesis that job stress has a negative correlation with safety behavior. Results show that the hypothesis is valid, and there is a negative correlation between job stress and safety behavior. In addition, finer results of the relationship between the six dimensions of job stress and safety behavior can be obtained. In summary, this study developed an improved stress scale for construction workers in China, and the proposed scale was validated by analyzing the data from an empirical study in Beijing.

## 1. Introduction

With the rapid increase of construction projects in the past several decades, the safety challenges in the construction industry have become increasingly large [1,2]. Although workplace safety has improved dramatically in the past few decades, accidents still occur in construction [3]. Compared to other industries, the research in safety on construction projects is often inadequate [4] because the profit margin is relatively low [5]. This leads to a higher accident rate in the construction industry [6]. The accident rate in construction is about three times as great as that of other industries, and the fatality rate is five times greater for construction compared to all industries [7]. The causes of accidents are usually categorized into three groups: technical failures, management issues, and human factors, in which unsafe behaviors are easily overlooked [8]. There are various reasons for unsafe behaviors, and job stress is one of the root causes. Many factors can influence job stress and safety behavior [9], such as safety culture, self-perceived fatigue, and personal characteristics. A study [9] found that self-perceived fatigue caused by job stress could directly affect safety behavior. Therefore, job stress can degrade the staff’s safety behavior through self-perceived fatigue.

Job stress refers to an extension of general stress: the nature of job stress is different from general stress in that job stress is specifically a result of work settings. In work settings, various factors can cause stress, such as the work task, the workplace, the job characteristics, role conflict, or worker capabilities [10]. Job stress threatens the health of the workers [11] and damages their job performance [12,13]. However, through questionnaire surveys, workers can measure their job stress to a certain extent by subjectively describing and rating the pressure. Studies [13,14,15,16,17,18] have shown that prolonged, high-level job stress can cause mental fatigue, resulting in awkward working posture and, consequently, unsafe behavior. The job stress of front-line construction workers is extraordinarily high due to the high complexity of their tasks, many of which are conducted in a harsh and dynamic environment or confined space [15]. Although there are many studies on job stress, covering the fields of coal, aviation [19], and sanitation [20], few of them are construction-related [21]. Sampson [22] et al. Gilboa [23] et al. and Sonnentag [24] et al. supported propositions that different job stress may have different relationships with various aspects of safety behavior. However, it has not been supported by enough follow-up studies. Thus, further investigation of the relationship between job stress and safety behavior is required. To study the relationship, an appropriate tool to measure job stress is required.

Therefore, this study aims to revise the existing job stress scale, which can be used for China’s construction projects to study the relationship between job stress and safety behavior. The rest of the paper is organized as follows: first, relevant research in the job stress and safety behavior is summarized in Section 2; second, the methodology of the stress scale development and validation is described in Section 3; third, the scale’s validation and the case of applying the developed scale to study the relationship between job stress and safety behavior in an empirical study are reported in Section 4 results; fourth, the developed scale and the application case of relationship analysis are discussed in Section 5; finally, the conclusions of the paper is summarized in Section 6.

## 2. Literature Review

In this section, a literature review was conducted to explore the structural dimensions of the job stress of construction workers, to enable the development of an appropriate job stress measurement questionnaire. The related works in the application case of job stress scale, the study of the relationship between job stress and safety behavior, is also summarized to propose the hypotheses to be tested in the case study.

### 2.1. Job Stress

The definition of stress is that it is the individual’s response to the imbalance between the demands of external events and the resources available to them to handle these demands [25]. Stress in work settings is defined as job stress [10]. Various factors can cause job stress, such as role ambiguity [26], role conflicts, role overload [27], job insecurity [28], job characteristics [27], employees’ ability [29], interpersonal safety conflicts [30], and safety restrictions [22]. Role ambiguity indicates that an individual’s role is unclear or inadequate in current information and resources. As the division of labor within the group is not clear, workers do not know the specific duties. Role conflict refers to the inconsistency between work performance expectations and performance evaluation standards. For example, a subordinate may receive multiple instructions from a superior at the same time without knowing which one to execute first. Alternatively, when a subordinate receives more than one command from a superior, he or she finds it difficult to execute both. Role ambiguity and role conflicts can lead individuals to an ambiguous position, and they may be confused about how to achieve the goal, what is right or wrong, and which superior feedback is effective. Interpersonal conflict refers to existing opinions, such as differences in equipment operations, causing conflicts between individuals and others in the organization [31]. To conclude, due to the negative effects of stress, they may have a negative impact on safety behavior.

Taking into account the different cultural backgrounds and knowledge levels of the construction workers, combined with the background of the Chinese construction industry, the initial version of the job stress scale is similar to the Occupational Stress Indicator [32], and the scale was revised using semi-structured interviews. Semi-structured interviews are used to modify the scale and then make it applicable to the area of construction projects in China. Therefore, this study proposed six dimensions of construction workers’ job stress, i.e., the job itself, role management, interpersonal relationships, organizational style, career development, and family-work conflict. The job itself refers to the job stress caused by factors such as heavy workload, short time, excessive responsibility, and unsafe conditions. Family-work conflict refers to job stress resulted from the lack of communication with the family due to a long-term job or the burden of low income; occupational development refers to the job stress caused by poor job stability; organizational style refers to the job stress caused by some not guaranteed rights or unreasonable system; interpersonal relationship refers to job stress resulted from unharmonious job relationship with colleagues or leaders, or not enough support and understanding of the leader; role management refers to the job stress generated by the uncertainty of their job responsibilities or conflict due to different job requirements.

### 2.2. Safety Behavior

Traditionally, safety behavior has been measured primarily by lagging indicators such as accident rates and mortality rates. However, these methods have limitations. They can form a skewed distribution by using accident frequency measurements. In addition, they cannot provide advanced warnings for pressure related accidents. Griffin and Neal [33] further proposed two sub-dimensions of safety behavior: safety compliance and safety participation. Similar to task performance, safety compliance refers to the core safety activities that need to be carried out by individuals to maintain workplace safety, including procedural compliance, wearing safety equipment, etc. Parallel to contextual performance, safety participation refers to employees voluntarily participating in safety activities or safety meetings, which is beneficial to the improvement of safety concerns and the organization’s safety program. Safety compliance belongs to in-role behavior, while safety participation is more voluntary, containing extra-role behavior. This study adopts the definition and components of safety behavior developed by Griffin and Neal.

### 2.3. Job Stress and Safety Behavior

The relationship between different job stress and two kinds of safety behavior (safety compliance and safety participation) may be different. This may be because when under job stress, the performance of safety compliance and safety participation may be both undermined, construction workers may try their best to first finish the required work (safety compliance) with limited time and energy, while having less time and energy to engage in participatory activities (safety participation). Thus workers under high job stress will reduce safety participation to a greater degree than safety compliance [34]. In addition, different job stress can have various influences on safety behavior, for example, Gilboa [23] et al. found that role ambiguity was most strongly negatively related to safety behavior than role conflict. Eatough [35] et al. proved that a stronger relationship existed between role conflict and safety participation than that between role conflict and safety compliance. To sum up, it needs further research on the relationship between different job stress and two kinds of safety behavior.

As job stress increases, the worker’s safety behaviors deteriorate accordingly. For instance, Research [36] has shown that job stress has a significant influence on safety behavior. According to a study of teachers’ burnout syndrome [37], weak interpersonal relationships can cause people to experience taedium vitae, causing mental fatigue which can increase the possibility of unsafe behavior. Based on the Effort-Reward Imbalance Model [38], employees consider career development as an important factor for themselves. In addition, because of the spillover effects of the safety climate [36], family and work can influence each other [37,39]. Some family issues may affect work by causing job stress [40]. When making a cognitive evaluation of the job stress environment, an individual experiences frustration, causing the individual to have negative emotional responses. The negative emotional response might eventually lead to unsafe behavior. Based on existing studies, safety behavior is measured in two dimensions, and job stress is measured in six dimensions. An increase in job stress in any one of the six dimensions might lead to lower safety participation and lower safety compliance. In this study, the authors will use the developed job stress scale to test the hypothesis that job stress will adversely affect safety behavior (safety compliance, safety participation).

## 3. Methodology

Through the literature review and interview process, the authors explored dimensions of the job stress of the construction workers, designed the job stress measurement questions, and cited the safety behavior measurement scale. Next, the authors interviewed the construction workers to verify the reliability of the items in the job stress questionnaire. The data on the job stress of the construction workers were obtained through the questionnaire survey. Finally, the authors used the data analysis method to study the relationship between the dimensions of the job stress and two kinds of safety behavior, and therefore demonstrate the applicability of the developed job stress scale in construction safety research.

### 3.1. Research Tools

To study the correlation between job stress and other factors, job stress must be quantitatively measured. Several tools [32,33,41] were developed to measure job stress. Among those tools, the Occupational Stress Indicator (OSI), developed by Cooper and Williams et al. [32], is one of the most frequently used to measure job stress. The scale includes six aspects, namely, the job itself, interpersonal relationship, role management, career and achievement, organization structure, and conflicts between families and works. The questionnaire developed by Karasek [41] is used to measure job control level and the psychological demand, and this questionnaire is divided into four sections: mission control, decision control, resource control, and physical job environment control.

Similar to job stress, safety behavior also needs to be measured quantitatively. Griffin and Neal [33] developed a questionnaire to assess the worker’s safety behavior in two dimensions, including the safety participation and safety compliance. In this questionnaire, six items are developed to measure the safety behavior, and the worker responds on a 5-point scale. This questionnaire for measuring safety behavior is the most widely used one and adopted by other researchers [38,42,43]. This scale is adopted in this study to measure safety behavior.

### 3.2. Semi-Structured Interviews

The existing two scales have been widely utilized, but their application fields are not quite consistent with the areas involved in this study (construction projects). Therefore, some modifications were needed through semi-structured interviews. Twenty construction workers were randomly selected to participate in the semi-structured interview. In this process, combining the working characteristics of construction workers, some basic questions were raised and then answered by the workers. For example, “Do you think the job you do will bring corresponding job stress? If so, what are the aspects of stress?” “Do you have interpersonal pressure with colleagues and leaders at the job?” The purpose is to modify the existing scale and then make it applicable to the area of construction projects.

### 3.3. Participants and Procedure

This study identified a construction project in Beijing for the questionnaire distribution. Before distributing the questionnaire to the workers, a briefing of the study was provided to the workers to address any potential concerns that the workers might have. The items in the questionnaire were also explained to the workers to make sure that the workers understood the questions correctly.

Similar to the Occupational Stress Indicator (OSI), the initial version of the job stress scale covers six dimensions (i.e., the job itself, role management, interpersonal relationships, career development, organization style, and family-work conflicts) but with different question items. There are 20 items in total, measured using a 5-level Likert Scale.

Similar to the safety behavior scale developed by Griffin and Neal [33], the initial version of the safety behavior scale covers two dimensions (i.e., safety participation and safety compliance) but with different question items. There are six items in total. The questionnaire items were rated on a 5-point Likert scale, ranging from 1 = “strongly disagree” to 5 = “strongly agree”. The higher the scores, the more the workers agree with the description. These two scales, as shown in Table 1 and Table 2, were distributed to the workers to fill out. Then, the collected data from valid questionnaires were analyzed to study the correlation between job stress and safety behavior using Statistical Product and Service Solutions (SPSS) 22.0 (International Business Machines Corporation, New York, NY, USA).

A total of 150 questionnaires were distributed, and 132 responses were received. The response rate was 88 percent. After removing eight invalid responses, the number of valid responses became 124, and the effective response rate was 83 percent. Among the 124 responses, 90% of the respondents were male, 34% of the respondents were at an age between 41 and 50, 44% of the respondents had 10–15 years working experience, 49% of the respondents had a high school degree, and 85% of the respondents were married.

## 4. Results

Using the 124 valid responses, the following analysis was conducted.

### 4.1. Factor Analysis of the Scale

The primary task of factor analysis is to extract and synthesize the overlapping parts of the original variables into factors. It requires a strong correlation between the original variables. Otherwise, if the original variables are independent of each other, the degree of correlation is very low. If there is information overlap, there is no common factor, and no factor analysis is needed. Therefore, before the factor analysis, KMO (Kaiser-Meyer-Oklin Measure of Sampling Adequacy) and Bartlett test of sphericity method are used to analyze whether the original variables correlate, meaning whether it is suitable for factor analysis. The KMO method indicates that the higher the value of the measure, the more common factors between the variables. The KMO and Bartlett tests are performed on the recovered working pressure gauge. The KMO value is 0.86 (between 0.8 and 0.9), and the significant level of Bartlett test is 0, indicating that the job stress questionnaire is suitable for factor analysis. The results of factor analysis using SPSS are presented in Table 3. The 20 items can be grouped into eight factors.

Through rotation component matrix of job stress questionnaire, eight factors are obtained. As the correlation of factor 7 and factor 8 is small, they are removed. The remaining six factors include, for the job itself (Factor #1), family-work conflict (Factor #2), career development (Factor #4), organizational style (Factor #3), interpersonal relationship (Factor #5) and role management (Factor #6).

As shown in Table 4, the cumulative interpretation rate of these six factors is 72.50%, indicating that these six types of job pressure can summarize 72.50% of the information contained in the original variables of the job pressure of construction workers. The construct validity is ideal. Among them, the job itself has the highest interpretation rate of 21.77%; the family-work conflict is 16.02%; the career development dimension is 14.54%; the interpretation rate of organizational style, interpersonal relationship, and role management is 7.26%, 6.94%, and 5.97%, respectively. It indicates that the job itself is the primary source of job stress for construction workers. The family-work conflict and career development factors are the less significant sources of job stress, while the three factors of organizational style, interpersonal relationship, and role management are not the leading causes of construction workers’ job stress.

### 4.2. Reliability Analysis of the Scale

The internal consistency of the scale was validated through Cronbach’s Alpha coefficient [44]. As shown in Table 5 and Table 6, the scales are reliable, as the values of Cronbach’s Alpha based on Standardized Items are above 0.7. Cronbach’s Alpha based on standardized items is a more reliable correlation value, and it eliminates system errors based on factor analysis.

### 4.3. Correlation Analysis of Job Stress and Safety Behavior

The overall relationship of construction workers’ job stress on safety behaviors was analyzed using the correlation analysis. The Pearson correlation between job stress and safety behavior is −0.27 with at a significance level of 0.05, indicating a negative correlation between job stress and safety behavior. In another word, the higher the job stress, the worse the safety behavior. This result aligns with the conclusions drawn in most of the early research, but it is different from the conclusions in recent research. The Correlation between the six dimensions of job stress and the two dimensions of safety behavior of construction workers was analyzed using correlation analysis, and the results are shown in Table 7.

## 5. Discussion

### 5.1. Findings and Implications

In this study, a job stress scale of the construction workers was developed, and the recovered job stress questionnaire was tested by KMO and Bartlett. The KMO sample measure was 0.86, which is suitable for factor analysis. After factor analysis, six dimensions of job stress are identified, including the job itself, family-work conflict, career development, organizational style, interpersonal relationship, and role management. These six dimensions of job stress can summarize 72.50% of the information contained in the original variables of the job stress. In addition, the Cronbach’s alphas of the Job Stress Scale were 0.74, which demonstrates its high internal consistency. Therefore, the applicability of the job stress scales was better.

Through the correlation analysis, it is found that the Pearson correlation between job stress and safety behavior is −0.27 with at a significance level of 0.05, indicating a negative correlation between job stress and safety behavior. In another word, the higher the job stress, the worse the safety behavior. This means that the proposed hypothesis is valid. There are also different levels of negative correlation between the six dimensions of job stress and the two dimensions of safety behavior. As Table 7 shown, it was found that the magnitude of the correlation between each dimension of job stress and safety behavior is different and can be roughly divided into three gradients, and the correlation coefficients indicate the ranking of the importance of different job stress dimensions to safety behavior.

The job itself was the greatest negatively related to safety behavior. The reason behind this may be that the construction workers devote all their effort to finish the jobs, and not much effort is allocated to job safety when the projects have tight schedules, high intensity, and heavy tasks. For some workers, they do not even have enough time for rest; at the same time, they are less likely to prioritize safety participation. Role management, organization style, and interpersonal relationships were the second lowest negatively related to safety behavior. This indicates that they have the second most significant negative impact on the safety behavior of construction workers. Safety role ambiguity refers to the poor cognition of an individual’s role that is related to the task required and its norms. Poor interpersonal relationships usually lead to job stress and burnout, or even unsafe behaviors [45]. Sampson [22] et al. examined the correlations between job stress and safety behavior (safety participation and safety compliance). They found that job stress was negatively related to safety participation, while only role management was negatively related to safety compliance. The present results demonstrated that safety participation would be impaired by improper role management. Thus, high safety role management is likely to reduce individuals’ safety compliance, which is largely related to the required safety role. Career development and family-work conflict were the third lowest negatively related to safety behavior. Workers with good safety practices should be given more job opportunities, and all workers must be encouraged not to perform unsafe operations. Employees’ families should also be fully engaged and understanding, so that workers will not be distracted by family issues at the job. One interesting finding from this study is that the magnitude of the negative correlation between each dimension of the job stress and safety participation is stronger than that between each dimension of job stress and safety compliance. The possible interpretation of the finding is that when workers are faced with job stress, their investment (e.g., energy and time) in jobs reduces. In this case, they may just concentrate on finishing the first necessary task, namely, the task falling within the realm of their ordinary roles, such as observing safety norms and procedures. In contrast, owing to energy or time limitations, they are less likely to be involved in participatory activities that are beyond their job requirements.

The findings are like a study case in Hong Kong. In construction, Leung, and Liang [21] et al. studied the complicated relationships between five job stressors, two types of stress (physical and psychological), safety behavior, and accidents, and found that safety behavior among construction workers was hampered by physical stress, and enhanced when appropriate supervisor support was provided. The study in Hong Kong classified job stress as psychological stress and physical stress, but the distinction was not made in the study, which the authors say does not affect the analysis of specific factors. There was a study on job stress of the dentist [46], which consider almost the same factor as the study in Hong Kong. However, interpersonal relationship and family-work conflict had not been taken into account in these two studies. This study expanded the scope of examination of job stress, which can have a more comprehensive understanding of the source of job stress and better control of job stress.

Considering the negative relationship between job stress and safety behavior, managers should be aware of high job stress that workers may experience and take measures to reduce it. Attention should be paid to two dimensions of job stress, especially job itself and role management. Construction organizations should be a reasonable arrangement of construction workers’ tasks and rest time, and overwork should be forbidden. There should also be a reasonable arrangement of workload, so as not to overload workers, taking responsibility for the security of the construction workers at the same time. Organizations should provide adequate safety equipment and create a good atmosphere to guarantee that workers can work in a safe environment and be fully absorbed in the job. In China, as the educational level of construction workers is generally low, unclear communication with foremen and colleagues may easily lead to ambiguous understandings of job responsibilities and objectives [47]. The organization should have clear instructions on what each employee should accomplish so that they can clearly understand their job. In addition, before the assignment of job tasks, management should reach a consensus to avoid the same worker receiving different job instructions. Therefore, before and during a task, it is essential for the project management team to provide safety training to all workers among different organizations and to explain specific safety requirements of each type of work [34], which may be helpful for role management. Safety training is especially needed in the Chinese construction industry as previous research has indicated inadequate awareness and training on safety within it [48]. When construction workers have either conflicts or estrangement with their colleagues or supervisors [18], or the company’s regulations cannot satisfy the workers’ needs, the workers may easily experience negative and suppressed emotions, which can negatively affect the workers’ safety norms. However, due to their low position in the industry, construction workers often receive inadequate feedback or attention from their supervisors and management personnel [47]. Then interpersonal relationships may be presented. Thus, to strengthen the relationship within workgroups, managers may carry out employee-friendly plans or create an open environment that allows workers to communicate work issues, including safety-specific problems.

### 5.2. Limitations and Future Research

Despite these contributions, this research has several limitations, which also inspire potential future research. First, the use of self-reporting measures may affect the reliability of the results and undermine the validity of the data. In this regard, all the measurement scales used in the survey were adopted following an extensive literature review. In addition, the Cronbach’s alphas for all factors were greater than 0.5, which demonstrates the reliability of the measures used. However, we still suggest future research on using multiple data sources for assessing construction workers’ safety behavior to minimize the possible problems of self-report. Second, only six dimensions of job stress were selected, although they are considered the most influential stress in previous studies. Further research will expand the scope of research on job stress. Furthermore, the magnitude of the negative correlation between each dimension of the job stress and safety participation is stronger than that between each dimension of job stress and safety compliance, which requires further examination. Thus, future research is suggested to explore a deeper relationship between job stress and two kinds of safety behavior. Third, semi-structured interviews are intended to modify the scale and then make it applicable to the area of construction projects, there are no results of semi-structured interviews reported or discussed. Future research can conduct a more in-depth analysis of it. Finally, the generalization of the current results still needs to be cautious, as the sample is mainly from Beijing of China and was collected from the construction industry. Thus, future research can expand the sample by including more areas and verified by undertaking several industrial case studies.

## 6. Conclusions

Construction workers usually work in complex physical environments amid various hazards such as poorly maintained equipment and unsafe machinery. This directly influences their stress levels and safety behavior. Given this situation, this study was conducted to develop the job stress scale and demonstrate its applicability by investigating the relationship between job stress and safety behavior on a construction work site in Beijing.

This study contributes to the current stress-management research by developing a reliable factor structure of construction workers’ job stress, including the job itself, family-work conflict, career development, organizational style, interpersonal relationship, and role management. The case study shows that the developed job stress can support the study on the relationship between job stress and safety behavior. The job itself has the most significant negative impact on the safety behavior of construction workers. Role management, organization style, interpersonal relationships, career development, and family-work conflict have a relatively low influence on safety behavior, compared to the job itself. Moreover, it was also found that the magnitude of the negative correlation between each dimension of the job stress and safety participation is stronger than that between each dimension of job stress and safety compliance.

## Figures and Tables

**Table 1:** Job stress original scale.

Dimensions	Items
Job itself	C 1. My job is very complicated, and there is a heavy workload C 2. Worry about personal safety at job C 3. I often job overtime in my job C 4. Great responsibility, afraid of accountability
Role management	C 5. I do not know much about my job C 6. My job has not been clearly explained and explained C 7. Sometimes I receive different job requirements from the job leaders C 8. Sometimes I am assigned to different positions at the same time
Interpersonal relationships	C 9. Conflict or unhappiness with colleagues C 10. Feel isolated at the job
Organization style	C 11. Lack of support from leadership C 12. Leaders were unwilling or unable to help me with my job problems C 13. Unit wage system is not reasonable C 14. The organization did not respond well to my performance
Career development	C 15. I am worried about my future career development C 16. The job stability is poor, worried that the unit cannot get the project after this project C 17. My rights are sometimes not protected
Family-work conflict	C 18. I feel that I have a heavy financial burden on my family C 19. The nature of the job requires separation and not enough responsibility to take care of the family C 20. Family members do not have enough understanding and support for job

**Table 2:** Safety behavior scale.

Dimensions	Items
Safety compliance	C 1. I use safe equipment to do my job C 2. I use the correct procedure to finish the job C 3. Try to be as safe as possible in my job
Safety participation	C 4. I take part in additional activities to improve workplace safety C 5. I volunteered to take part in activities to improve workplace safety C 6. I volunteered to raise the security level of the organization

**Table 3:** Rotation component matrix of job stress questionnaire.

Factor	Items	Factor							
		#1	#2	#3	#4	#5	#6	#7	#8
Factor #1	C3	0.87	−0.02	0.10	−0.07	0.07	−0.12	0.10	−0.08
	C4	0.82	−0.08	0.10	−0.07	0.17	0.27	−0.20	−0.11
	C1	0.80	−0.02	0.08	−0.11	0.31	0.31	0.06	0.07
	C2	0.78	−0.11	0.15	0.14	−0.15	0.21	0.10	0.11
Factor #2	C19	0.20	0.79	0.17	−0.04	−0.10	0.18	0.22	0.13
	C18	0.09	0.74	−0.16	−0.22	0.06	0.03	−0.31	−0.39
	C20	−0.26	0.68	0.34	0.06	0.04	0.13	−0.24	−0.19
Factor #3	C14	−0.14	0.25	0.96	0.32	−0.45	−0.06	−0.14	0.04
	C13	0.16	0.28	0.92	0.20	−0.14	0.20	0.02	0.10
Factor #4	C16	0.15	0.16	0.18	0.84	−0.29	0.15	0.12	0.20
	C15	−0.28	−0.61	0.17	0.78	0.24	0.09	−0.02	0.24
	C17	−0.06	0.11	−0.03	0.63	0.05	0.08	0.06	0.19
Factor #5	C9	−0.24	−0.12	−0.18	0.13	0.75	−0.15	0.03	−0.33
	C10	0.18	0.04	0.26	−0.11	0.70	0.28	0.12	−0.23
	C11	−0.01	0.20	0.04	0.48	0.58	0.17	−0.12	0.22
Factor #6	C7	−0.07	0.08	−0.06	0.13	0.02	0.73	0.32	0.25
	C6	−0.22	0.53	−0.13	0.01	0.07	0.70	−0.07	−0.12
	C8	0.11	−0.47	0.08	0.16	−0.12	0.66	0.11	0.01
Factor #7	C5	0.23	−0.05	−0.15	0.11	0.92	0.01	0.78	0.24
Factor #8	C12	0.29	−0.03	−0.17	−0.11	0.75	0.12	−0.02	0.71

**Table 4:** Total variance explained by the job stress questionnaire.

Factor#	Initial Eigenvalue			Extract Square Sum Loading		
	Total	Variance %	Accumulation %	Total	Variance %	Accumulation %
1	3.92	21.77	21.77	3.92	21.77	21.77
2	2.88	16.02	37.79	2.68	16.02	37.79
3	2.62	14.54	52.39	2.32	14.54	52.39
4	1.31	7.26	59.59	7.26	10.03	59.59
5	1.25	6.94	66.54	6.94	6.94	66.54
6	1.07	5.97	72.50	5.97	5.97	72.50

**Table 5:** Job stress scale reliability statistics.

Variable	Cronbach’s Alpha	Number of Items
Job itself	0.79	4
Family-work conflict	0.73	3
Career development	0.84	2
Organization style	0.70	3
Interpersonal relationship	0.68	3
Role management	0.70	3
Questionnaire	0.73	18

**Table 6:** Safety behavior scale reliability statistics.

Variable	Cronbach’s Alpha	Number of Items
Safety compliance	0.82	3
Safety participation	0.64	3
Questionnaire	0.72	6

**Table 7:** Correlation analysis of each dimension (*n*= 124).

The Variable		Job Itself	Role Management	The Career Development	Organization Style	Interpersonal Relationship	Family-Work Conflict
Safety compliance	Pearson correlation	−0.31 **	−0.21 *	−0.10	−0.25 *	−0.27 *	−0.17
	Significance (bilateral)	0.00	0.04	0.13	0.04	0.04	0.09
Safety participation	Pearson correlation	−0.35 **	−0.28 **	−0.12	−0.29 **	−0.29 *	−0.19
	Significance (bilateral)	0.00	0.004	0.08	0.00	0.03	0.12

* A significant correlation was found at the 0.05 level (bilateral); ** A significant correlation was found at the 0.01 level (bilateral).
